# Editorial: Structure, Isotypes, Targets, and Post-translational Modifications of Immunoglobulins and Their Role in Infection, Inflammation and Autoimmunity

**DOI:** 10.3389/fimmu.2020.01761

**Published:** 2020-08-07

**Authors:** Jean Harb, Bridget S. Wilson, Sylvie Hermouet

**Affiliations:** ^1^Centre de Recherche en Transplantation et Immunologie UMR1064, Inserm, Université de Nantes, Nantes, France; ^2^CRCINA, Inserm, Université de Nantes, Université d'Angers, Nantes, France; ^3^Laboratoire de Biochimie, CHU de Nantes, Nantes, France; ^4^University of New Mexico Comprehensive Cancer Center, Albuquerque, NM, United States; ^5^Department of Pathology, University of New Mexico School of Medicine, Albuquerque, NM, United States; ^6^Laboratoire d'Hématologie, CHU de Nantes, Nantes, France

**Keywords:** immunoglobulins, glycosylation, targets, inflammation, infection, autoimmunity

Infection, autoimmunity, and cancer are accompanied by inflammation, which may alter the structure and function of immunoglobulins (Ig) and consequently, their pathogenicity (1–3). In addition, the isotype also influences the pathogenicity of Igs (4). During Dengue virus infection, the removal of core fucose residues selectively enhances the affinity of IgG for Fragment crystallizable (Fc)γIIIa receptors, leading to increased antibody-dependent cell mediated cytotoxicity (ADCC) and decreased complement dependent cytotoxicity (CDC) (5). In patients infected by the human immunodeficiency virus (HIV), anti-gp120 antibodies are less galactosylated and sialylated in asymptomatic, long-term non-progressors, compared to symptomatic patients (6). The Fc domain of IgGs can trigger pro- or anti-inflammatory responses and there is abundant evidence that carbohydrates attached to the IgG Fc domain are essential for IgG function (7–9). The pro- or anti-inflammatory function of IgGs is mediated by different affinities for activating FcγRs (FcγRI, RIIa, RIIIa, and RIIIb) and inhibiting FcγRIIb expressed by immune cells (10–12). A high level of sialylation of the IgG Fc fragment decreases ADCC potential through low affinity for activating receptors and conversely, bisecting N-acetylglucosamines on the Fc fragment are pro-inflammatory and enhance ADCC (13–16). In autoimmune diseases, such as rheumatoid arthritis, patients show low levels of IgG Fc sialylation, while increased IgG sialylation is associated with remission (17, 18). Thus, the glycosylation level of IgGs may explains their “protective” action. Similarly, Ig glycosylation plays an important role in IgA nephropathy, where IgA1s are deficient in galactose and not correctly cleared by anti-IgA1 antibodies (19).

The targets of Igs are also of increasing interest in human pathology, and important antigenic drivers are being discovered in monoclonal gammopathies of undetermined significance (MGUS) and myeloma, a blood cancer (20–26). The immunogenic glucolipid glucosylsphingosine (GlcSph)—also called lysoglucosylceramide (LGL1)—and infectious pathogens including Epstein-Barr virus (EBV) and hepatitis C virus (HCV), were recently shown to be the targets of monoclonal IgGs in MGUS and myeloma (20–26). Monoclonal IgGs bear very low levels of sialylated glycans, which suggests a pro-inflammatory state and reinforces the notion that chronic antigenic stimulation and an abnormal immune response contribute to the pathogenesis of MGUS and myeloma (27). Importantly, therapies aiming at reducing the target of the monoclonal Ig can be proposed to patients. Recent reports described the beneficial effect for patients who presented a GlcSph (LGL-1)-reactive monoclonal Ig and who received treatments that reduced the level of immunogenic glucolipid; a clear reduction in monoclonal Ig was obtained for two patients (28). Similarly, antiviral treatment benefited both MGUS patients and myeloma patients with a monoclonal Ig that targeted HCV (29, 30).

This collection constituted of 13 original articles, 1 case report and 2 reviews from 112 authors, is divided into three sections. The first section presents recent knowledge on the variability in structure and isotype of Igs in clinical contexts. The second section is devoted to the targets and post-translational modifications of Igs in specific pathological contexts. The final section describes the consequences of Ig variability and targets in terms of pathogenicity and interest for the diagnosis, prognosis, monitoring, and treatment of patients.

## Structure and Isotypes of Immunoglobulins

The structure of Igs influences their function and also their fate (half-life, for instance) and subsequently, their efficacy. In this regard, the studies of Deveuve et al. highlight the importance of the hinge region of Igs, particularly for the development of new therapeutic monoclonal antibodies (TmAbs) (Deveuve et al.). They analyzed the proteolytic cleavage of the hinge region of IgG, which may occur by proteases of the microenvironment, including matrix metalloproteinase 12 (MMP12) or bacterial (Streptococcus piogenes) Ig-degrading enzymes (IdeS) and represents an escape mechanism to treatment by TmAbs. The authors compared the cleavage of 8 TmAbs of different isotypes and found the IgG2 TmAb more protease resistant than IgG1 and IgG4 TmAbs, and variable IdeS-sensitivity among IgG4 and IgG1 TmAbs. They propose that the variability in the cleavage sensitivity/resistance balance among IgG1 and IgG4 TmAbs results in part from characteristics of the Fab region (Deveuve et al.). They also show that a single cleavage of IgG1 TmAbs greatly decreases their affinity for FcγRIIIa and ability to induce FcγRIIIa-dependent functional responses from NK cells.

Allergy is dependent on the IgE isotype. Koning et al. studied IgE VDJ sequences from allergic patients, and compared them to the IgE repertoire from healthy, non-atopic individuals. They report that IgE repertoires were highly oligoclonal with preferential usage of certain IGHV genes. IgE sequences had no clonal relationship with the other isotypes, carried more somatic mutations than IgM but fewer than IgG and IgA. Thus, in healthy individuals, the mutational burden of IgE suggests an origin through direct class-switching from the IgM repertoire, and presumably low affinity for antigens.

The risk of transplant rejection is also affected by the isotype of antibodies developed by patients against human leukocyte antigens (HLA). Navas et al. describe the results of the analysis of 1,285 anti-HLA antibodies identified in serum samples from 20 highly HLA-sensitized patients, and report that 36.8% of anti-HLA antibodies were C1q-binding. They found a strong association between C1q-binding ability and IgG1 strength, whereas weak or non-C1q-binding IgG2 and IgG4 subclasses were common. They conclude that the IgG1 subclass best correlates with the C1q-binding ability of anti-HLA antibodies.

## Targets and Post-translational Modifications of Immuno-Globulins in Specific Pathological Contexts

### Targets of Immunoglobulins

The antigenic targets of pathological Igs are relatively well-known in the context of allergy and auto-immune diseases. In the context of B-cell malignancies, the main objective is to eliminate the malignant clone and most studies aim to characterize tumoral cells and uncover the mechanisms of their resistance to treatments. Consequently, the antigenic targets of the Igs produced by malignant B-cell clones are rarely studied. Yet there is mounting evidence that chronic antigen stimulation as an important pathogenic mechanism in the development of B-cell malignancies. For instance, patients with somatically–mutated (antigen-driven) chronic lymphocytic leukemia (CLL) have a more favorable clinical course than other CLL patients (31, 32). Evidence in favor of chronic antigen stimulation has also been reported in MGUS and in myeloma (20–26). CLL-associated antigens appear to be mostly autoantigens, notably cytoskeleton components, or autoantigens found in apoptotic cells and bacteria (33–35). Cases of virus (HCV), HIV-driven CLL, MGUS or myeloma appear to be relatively rare (36). In MGUS and myeloma, the targets of monoclonal IgG reported include viruses (>25% cases, predominantly EBV and more rarely, herpes virus simplex (HSV) and HCV) and glucolipids, particularly GlcSph (LGL1) (~15% cases) or associated enzymes (22–26). Using a new assay based on the protein micro-array technology, Bosseboeuf et al. report that the purified monoclonal Ig from 42% of IgA MGUS and myeloma patients recognize EBV EBNA-1, HCV or LGL1. Altogether, a pathogenic model of antigen-driven disease may be valid for about half of CLL, MGUS, and myeloma cases. This model offers new therapeutic approaches: in addition to current therapeutic protocols aimed at eliminating the malignant clone, one can envision therapies designed to reduce or suppress the antigen responsible for disease initiation, i.e., the target of the patient's monoclonal Ig, whenever the target can be identified (28–30).

Unfortunately, the identification of the targets of pathogenic human IgG and IgA for diagnosis and therapeutic purposes is still not possible outside research laboratories. Similarly, the targets of IgMs from healthy individuals are rarely investigated and remain poorly known. To facilitate the study of human IgM antibodies, which are characterized by polyspecificity and autoreactivity, Pashov et al. propose a new peptide array consisting of 594 mimotopes that reflect the common IgM repertoire of 10,000 healthy donors.

### Post-translational Modifications and Function of Immunoglobulins

To interpret the results of IgG functional studies, the conditions of preparation and purification of Igs are of great importance. Lopez et al. report that low pH exposure during IgG purification may result in aggregates that abnormally and avidly bind Fcγ receptors. These authors compared Protein G purification of IgG (at low pH) with an immunoaffinity method which elutes IgG at physiological pH, and investigated several factors known to impact Fc functionality and influence FcγR binding, including IgG subclass, *N*-glycosylation, aggregation, and conformational changes. They show that low pH elution of IgG enhances their recognition of FcγRs, and increases IgG aggregation. Thus, differences in IgG purification methods may explain the poor reproducibility of studies of Fc-mediated antibody functions.

The important role played in immunity by the N-linked glycosylation of the Fc region of Igs is well-established but the function of N-linked glycosylation of the variable domains of Igs is less well-known. In their review, Vletter et al. report that N-linked glycans are present on autoantibodies, notably in rheumatoid arthritis, and in patients with B-cell follicular lymphoma (FL). N-linked glycosylation of the variable domain of Igs may confer a selective advantage, through interaction with lectins and/or microbiota. They analyzed the characteristics of autoantibodies and those of Igs from FL patients and healthy donors and found differences in variable domain glycan distribution, frequency and glycan composition, which led them to propose a classification of diseases according to the specific Ig variable domain glycosylation patterns observed in patients.

Sialylation may also modify therapeutic IgGs. Shaffert et al. analyzed changes in human intravenous IgG (IVIg) sialylation upon injection in mice deficient in B cells or lacking the sialyltransferase 1, which catalyzes the addition of α2,6 linked sialic acid residues and conclude that the glycosylation of therapeutic IgGs is stable *in vivo* Schaffert et al.. Only a very small fraction of IgGs acquired sialic acid structures, mostly in the Fab portion, not in the Fc portion.

## Consequences in Terms of Diagnosis, Prognosis, Monitoring of Patients, and Therapy

The detection of monoclonal or/and polyclonal Igs with identified targets is becoming of increasing importance in different clinical contexts, including severe infection, chronic inflammation, and certain blood cancers. It is also necessary to better understand the effects of therapeutic monoclonal antibodies used in the clinic, alone or associated with other treatments.

### Infection

The interest of understanding the Ig response of patients in context of acute infection is illustrated by the report by Bloomfield et al., who describe the case of an infant who presented with a normal C-reactive protein (CRP) level despite severe septic shock following *Staphylococcus aureus* infection, with clear biological evidence of systemic inflammation. Bloomfield et al. suspected a defect in the interleukin-6 (IL-6)/CRP axis, which was confirmed by the presence of neutralizing anti-IL-6 autoantibodies in the child's serum. These findings are of importance since clinical interference of IL-6 signaling, for instance with IL-6 receptor-targeting therapeutic antibodies such as tocilizumab or sarilumab, may alter IL-6-mediated innate immune responses and compromise host resistance to infections.

The complex role played by Igs in chronic infection is illustrated in the review by McLean et al. on the mechanisms of tuberculosis reactivation in individuals with comorbidities. They describe the Ig responses to *Mycobacterium tuberculosis* of patients with chronic conditions, such as co-infection with HIV, diabetes or kidney diseases, where inflammation may facilitate tuberculosis re-activation. McLean et al. propose that inflammatory IgG profiles may be important biomarkers for the detection of progressive tuberculosis. More studies are needed to distinguish inflammatory antibody profiles that are the consequences of co-morbidities from those that contribute to the reactivation of tuberculosis.

Regarding severe viral infections, neutralizing antibodies hold great promise both for antibody-based therapeutic intervention and for vaccine design. This is illustrated by the paper by Gao et al., who applied next-generation sequencing (NGS) to probe the development of ZK2B, a potent E DIII-specific antibody protective against Zika virus (ZIKV), isolated from a convalescent individual. The NGS-derived, germline-like ZK2B10 somatic variants neutralized ZIKV and protected mice from ZIKV challenge, without cross-reactivity with Dengue virus. Site-directed mutagenesis identified residues essential to the functional maturation of ZK2B10. The repertoire and lineage features unveiled in this study should help elucidate the developmental process and protective potential of anti-ZIKV antibodies.

### Auto-Immunity

Gray et al. report on a rapidly progressive glomerulonephritis reproducing the auto-immune Goodpasture's disease (GP), caused by anti-glomerular basement membrane (GBM) antibodies developed after allogenic haematopoietic stem cell transplantation (HSCT). In GP, autoantibodies bind neoepitopes formed upon disruption of the structure of α345 NC1 hexamer, a critical domain of α345 collagen IV scaffolds. Upon hexamer disruption, α3 and α5 NC1 subunits become immunogens. Gray et al.'s is the first report of allo-incompatability and antigenic specificity in anti-GBM disease after allogenic HSCT. Both the patient and donor presented with the Goodpasture's susceptibility HLA-allele DRB1^*^1501, and the patient's anti-GBM antibodies recognized the E_A_ epitope of the α3 NC1 monomer of collagen IV. Auto-antibody binding to native α345 NC1 hexamer was minimal, and there were no polymorphic differences between the donor's and recipient's collagen IV genes. The authors conclude that their patient's was a case of classical GP disease, the anti-GBM antibodies emerging post transplantation from the donor immune system. This hypothesis is supported by the finding that native a345 NC1 hexamer was not pathogenic in an animal model of GP disease, whereas immunization with dissociated hexamers induced glomerulonephritis (Gray et al.).

Another example is provided by systemic lupus erythematosus (SLE), where Zhang et al. investigated the role of IgG in spleen inflammation. They report that lupus IgGs are important pathological factors involved in the initiation of inflammation and further germinal center (GC) and plasma cell formation. Macrophages of the splenic marginal zone were dispensable for the GC response induced by lupus IgG, while red pulp macrophages were important for GC responses. Furthermore, lupus IgGs promoted inflammation and GC formation through the macrophage-mediated secretion of TNF-α. Interestingly, Syk inhibitor treatment suppressed the changes in the histopathology of the spleen induced by lupus IgGs.

## Conclusion

Accumulating new knowledge of the structure, targets, and glycosylation of pathogenic Igs should rapidly translate into improved diagnosis and treatments for patients suffering from acute or chronic infection, chronic inflammation, autoimmunity, or B-cell malignancies.

## Author Contributions

All authors listed have made a substantial, direct and intellectual contribution to the work, and approved it for publication.

## Conflict of Interest

The authors declare that the research was conducted in the absence of any commercial or financial relationships that could be construed as a potential conflict of interest.
